# Comparison of Efficacy and Safety Between Standard, Accelerated Epithelium-Off and Transepithelial Corneal Collagen Crosslinking in Pediatric Keratoconus: A Meta-Analysis

**DOI:** 10.3389/fmed.2022.787167

**Published:** 2022-03-17

**Authors:** Yuanjun Li, Ying Lu, Kaixuan Du, Yewei Yin, Tu Hu, Yanyan Fu, Aiqun Xiang, Qiuman Fu, Xiaoying Wu, Dan Wen

**Affiliations:** ^1^Eye Center of Xiangya Hospital, Hunan Key Laboratory of Ophthalmology, Xiangya Hospital, Central South University, Changsha, China; ^2^National Clinical Research Center for Geriatric Disorders, Xiangya Hospital, Central South University, Changsha, China

**Keywords:** pediatric keratoconus, corneal crosslinking, epithelium-off, accelerated epithelium-off, transepithelial, visual acuity, Kmax

## Abstract

**Purpose:**

The purpose of the study is to compare the efficacy of standard epithelium-off CXL (SCXL), accelerated epithelium-off CXL (ACXL), and transepithelial crosslinking CXL (TECXL) for pediatric keratoconus.

**Methods:**

A literature search on the efficacy of SCXL, ACXL, and TECXL [including accelerated TECXL (A-TECXL)] for keratoconus patients younger than 18 years was conducted using PubMed, Cochrane Library, ClinicalTrials.gov, and EMBASE up to 2021. Primary outcomes were changes in uncorrected visual acuity (UCVA) and maximum keratometry (Kmax) after CXL. Secondary outcomes were changes in best-corrected visual acuity (BCVA), mean refractive spherical equivalent (MRSE), and central corneal thickness (CCT). Estimations were analyzed by weighted mean difference (WMD) and 95% confidence interval (CI).

**Results:**

A number of eleven identified studies enrolled 888 eyes (SCXL: 407 eyes; ACXL: 297 eyes; TECXL: 28 eyes; A-TECXL: 156 eyes). For pediatric keratoconus, except for a significant greater improvement in BCVA at 24-month follow-up in SCXL (WMD = –0.08, 95%CI: –0.14 to –0.01, *p* = 0.03, I^2^ = 71%), no significant difference was observed in other outcomes between the SCXL and ACXL groups. SCXL seems to provide greater changes in UCVA (WMD = –0.24, 95% CI: –0.34 to –0.13, *p* < 0.00001, I^2^ = 89%), BCVA (WMD = –0.09, 95% CI: –0.15 to –0.04, *p* = 0.0008, I^2^ = 94%), and Kmax (WMD = –1.93, 95% CI: –3.02 to –0.85, *p* = 0.0005, I^2^ = 0%) than A-TECXL, with higher incidence of adverse events.

**Conclusion:**

For pediatric keratoconus, both SCXL and ACXL appear to be comparable in the efficacy of visual effects and keratometric outcomes; SCXL seems to provide greater changes in visual and pachymetric outcomes than A-TECXL.

## Introduction

Keratoconus is a corneal ectatic disorder characterized by asymmetric progressive conical steepening and thinning (1). The prevalence of keratoconus varies among populations with an estimate of 1/2,000 worldwide (2). In pediatric population, the prevalence of pediatric keratoconus is reported to be higher from 1/375 to 1/2,000 (3). Keratoconus commonly presents in the second decade and progresses until the third or fourth decade of life; compared with adults, pediatric keratoconus is more severe with a higher risk of deterioration and faster progression (4, 5). The clinical characteristics of keratoconus include progressive loss of vision and increasing irregular astigmatism, which results from a more conical shape in thinning and steepening cornea (6). Vision loss is often caused by myopia and irregular astigmatism, and in rare cases, the scarring with or without rupture of Descemet’s membrane and corneal edema. In comparison with adults, keratoconus in children appears more centrally located ectatic cornea and often progresses asymmetrically, which leads to good binocular visual performance until both eyes are affected. These factors may contribute to a late seeking in medical care and more deteriorated visual function in pediatric patients (3). Visual impairment in keratoconus severely affects educational, economical, and social development, which may decrease patients’ quality of life. Thus, early and prompt intervention to halt the progression and improve visual quality is very important.

Multiple factors at cellular, physiological, biomechanical, and genetic levels contribute to the progression of keratoconus, and main changes among them are alterations in collagen fiber, which includes the gradual loss of fibril orientation, and weaken intra- and interfibrillary collagen crosslinks (7, 8). Based on an interaction between riboflavin (as a photosensitizer) and ultraviolet A (UVA) radiation, CXL aims to mitigate the progression of the disease by strengthening rigidity of corneal stroma and avoid the need for corneal transplantation (9, 10).

Although previous clinical trials have studied the postoperative efficacy of CXL in pediatric keratoconus and several have compared two or three protocols (11, 12), none of them provided comprehensive comparison of SCXL, ACXL, and transepithelial CXL (TECXL and A-TECXL). The small sample sizes in single study cast doubt on the validity of their conclusions. Meta-analysis of the comparison between epithelium-off and transepithelial CXL in adult patients has suggested that SCXL and TECXL might provide comparable effects on visual and pachymetric outcomes after surgery (13). A recent meta-analysis of CXL in pediatric patients included 21 studies and determined the efficacy and safety, but did not focus on the comparison between different protocols (14). Hence, we conducted a meta-analysis to compare the efficacy of SCXL with ACXL and transepithelial CXL in pediatric keratoconus.

## Materials and Methods

### Evidence Acquisition

This meta-analysis was performed according to the Preferred Reporting Items for Systematic Reviews and Meta-Analyses (PRISMA checklist guidelines) (15).

### Search Strategy and Study Selection

**FIGURE 1:**
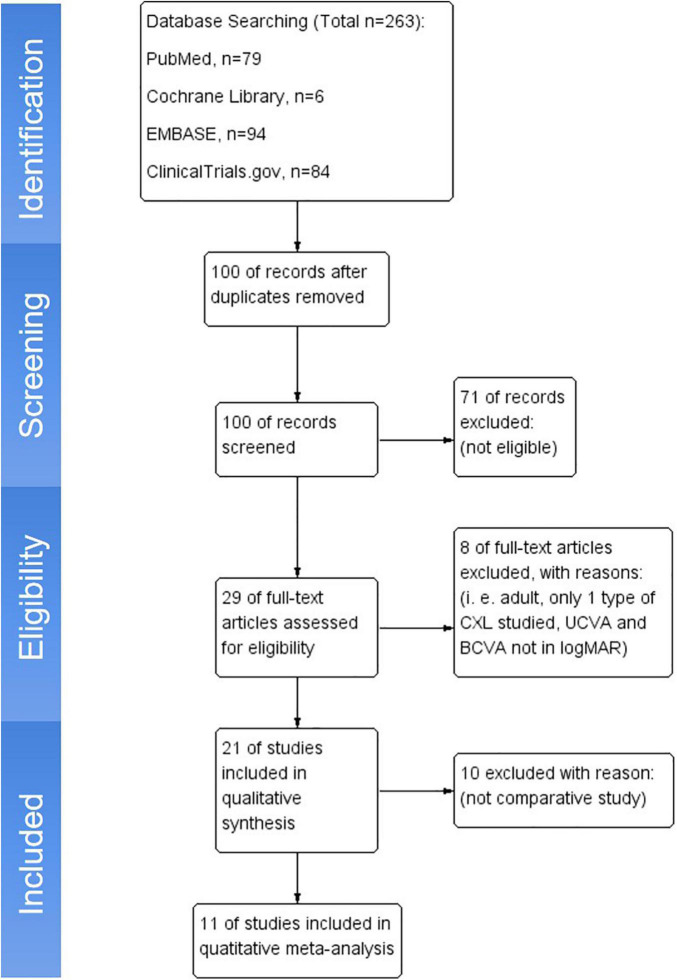
Flow diagram of the study selection.

A comprehensive literature search was conducted in several databases that include PubMed, Cochrane Library, ClinicalTrials.gov, and EMBASE from earliest available dates to March 2021 with language striction in English. The keywords “keratoconus,” “pediatric,” and “corneal collagen crosslinking” OR “CXL” were searched. The related-articles function was also applied to broaden results from the search engine. Reference lists from the publications were also checked for relevant studies. Detailed search strategies are provided in Supplementary Table 1. Retrieved papers were screened by two authors (YJL and YY) independently, and duplicated studies were removed. Using the inclusion and exclusion criteria described below, the papers were then assessed for meta-analysis. The literature search and selection are shown as a flowchart (Figure 1).

### Inclusion and Exclusion Criteria

Articles were retained if they met the following inclusion criteria: (1) observational comparative studies; (2) focused on keratoconus in patients aged 18 or younger; (3) involved at least two types of CXL protocols that include SCXL, ACXL, or transepithelial CXL; (4) outcomes that contain at least UCVA (transferred to the log minimum angle of resolution, LogMAR), BCVA, Kmax, and CCT. Articles were excluded if any of the following conditions existed: (1) studies with inadequate information for calculating data on outcomes and (2) duplicated report. If multiple studies by the same research team derived from different populations were available, all of them were deemed eligible and included in the meta-analysis.

### Data Extraction

Two authors (YLi and YLu) extracted data independently with a standardized form. The following information was retrieved in all the included publications: first author name, year of publication, country, sample size of patients and eyes, mean age, gender, study design, type of CXL protocol, Amsler–Krumeich stage, follow-up time, UCVA, BCVA, MRSE, Kmax, and CCT at the observation point (at specific timepoints or the last follow-up). Changes in the outcomes before and after CXL were calculated (postoperative value deducting preoperative value) (16). Any discrepancies in data extraction or disagreements in the data were resolved by discussion and reassessment with the senior author (DW).

### Quality Assessment

The risk of bias of the included RCTs was evaluated using the Cochrane risk of bias tool, which contained seven domains: random sequence generation, allocation concealment, blinding of participants and personnel, blinding of outcome assessment, incomplete outcome data, selective reporting, and other sources of bias (Supplementary Figure 1) (15). The risk of bias in each domain was labeled as low (green), unclear (yellow), or high risk (red) for each study by two authors independently. The quality of the non-RCTs was assessed with the Newcastle–Ottawa Scale (NOS) from 0 (lowest quality) to 9 (highest) (Supplementary Table 2) (17).

### Statistical Analysis

Meta-analysis was performed using Review Manager version 5.3.5 (Cochrane Collaboration, London, United Kingdom). Continuous variables were compared using the WMD. Pooling estimates and their 95% confidential intervals (CI) were calculated. The fixed-effect model was applied, and heterogeneity was quantified using the I^2^ value, which represents the percentage of the total variation among studies. Cochrane Q-test *p* > 0.1 was considered as no significant heterogeneity, and the random-effects model was used to calculate pooling estimates and address within- or between-study variances. For a clear visualization, forest plots were produced. An I^2^ value of 25–50%, 50–75%, >75% was defined as low, moderate, and high heterogeneity, respectively. Statistically significance was measured by a *p* < 0.05. Analyses were stratified by the types of CXL protocol and follow-up time. Potential publication bias was evaluated by examining the symmetry of funnel plots.

## Results

### Study Selection

The flow diagram of the study selection is illustrated in Figure 1. Initially, a total of 263 articles were retrieved from databases. After removing the duplicates, 100 potential papers were left, and the titles and abstracts were reviewed. Among them, 79 articles were excluded because of irrelevant topics. Full texts of the remaining 21 papers were assessed in their entirety, and 10 articles were excluded as no comparative data or not suitable for analysis. Eventually, 11 articles that provided detailed quantitative data were included in this meta-analysis.

### Characteristics of the Included Studies

Details of characteristics of included 11 studies are summarized in Table 1. The studies were published between 2013 and 2020, totally examining 888 eyes from 597 pediatric patients. The follow-up time of each research was ranging from 6 to 36 months. Two studies were RCTs (18, 19), whereas the others were CNSs (20–28). Each study compared the UCVA, BCVA, MRSE, Kmax, and CCT of different CXL protocols. The surgical procedures of the included studies were either SCXL, ACXL, TECXL, or A-TECXL (Supplementary Table 3). There were 11 included studies that involve the application of SCXL, 7 studies that involve ACXL, and 5 studies that employ transepithelial CXL. As only 7 studies at most were included in the meta-analysis, potential publication bias was not examined.

**TABLE 1:** Characteristics of all included studies in the meta-analysis.

References	Country	Inclusion criteria*	Study design	Follow-up (months)	Mean age (SD) in years	% male	Surgery protocols (No. of eyes/patients)			
							SCXL	ACXL	TECXL	A-TECXL
Baenninger et al. (21)	Switzerland	Stage 1–2	CNS	12	SCXL: 16.31 (1.78) ACXL: 15.54 (2.15)	77%	39/31	39/30	–	–
Eissa and Yassin (19)	Egypt	Stage 1–2	RCT	12, 24, 36	12.3 (2.4)	NA	34/34	34/34	–	–
Iqbal et al. (18)	Egypt	Stage 1–3	RCT	6, 12, 24	14.36 (2.11)	49.26%	91/46	92/46	–	88/44
Nicula et al. (20)	Romania	Stage 1–4	CNS	12, 24, 36, 48	SCXL: 16.43 (1.28) ACXL: 16.77 (1.53)	SCXL: 64.9% ACXL: 74.1%	37/37	27/27	–	–
Sarac et al. (26)	Turkey	Stage 1–2	CNS	6, 12, 24	SCXL: 15 (0.30) ACXL: 14.92 (0.34)	SCXL: 72.5% ACXL: 71.5%	38/29	49/35	–	–
Turhan et al. (27)	Turkey	Stage 1–2	CNS	24	SCXL: 15.7 (1.6) ACXL: 16 (1.7)	NA	26/17	22/17	–	–
Eraslan et al. (22)	Turkey	Stage 1–3	CNS	24	SCXL: 15.5 (1.7) TECXL: 15.4 (1.7)	48.1%	18/12	–	18/15	–
Henriquez et al. (24)	Peru	Stage 1–2	CNS	6, 12	SCXL: 13.2 (NA) A-TECXL: 14.9 (NA)	60.8%	25/NA (total 51)	–	–	36/NA (total 51)
Henriquez et al. (25)	Peru	Stage 1–2	CNS	12, 60	SCXL: 13.2 (2.6) A-TECXL:14.6 (2.1)	55.38%	46/NA (total 65)	–	–	32/NA (total 65)
Amer et al. (28)	Egypt	Stage 1–2	CNS	36	SCXL: 15.3 (2.0) ACXL: 15.2 (2.5)	SCXL: 38.9% ACXL: 41.2%	34/18	34/17	–	–
Magli et al. (23)	Italy	Any stage	CNS	3, 6, 12	15.2 (1.7)	73.3%	23/19	–	16/11	–

SCXL, standard epithelium-off CXL; ACXL, accelerated epithelium-off CXL; TECXL, transepithelial corneal CXL; A-TECXL, accelerated transepithelial corneal CXL; CNS, comparative non-randomized study; RCT, randomized controlled trial; NOS, Newcastle-Ottawa Scale. *Amsler–Krumeich stage.

### Comparative Effectiveness of SCXL and ACXL

#### Primary Outcomes

**FIGURE 2:**
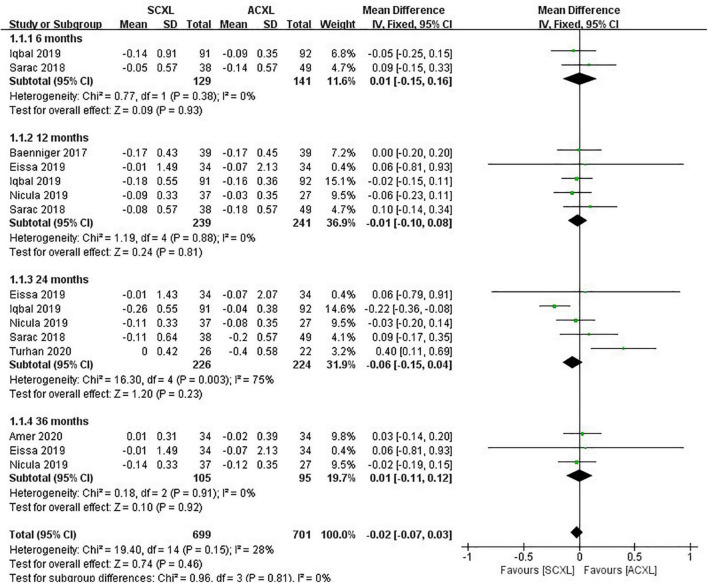
Forest plot of the change in UCVA (ΔUCVA) of SCXL and ACXL.

Uncorrected visual acuity: Overall, there was no significant difference in the change in UCVA between SCXL and ACXL through the follow-up (WMD = -0.02, 95% CI: –0.07 to 0.03, *p* = 0.46, I^2^ = 28%, Figure 2).

**FIGURE 3:**
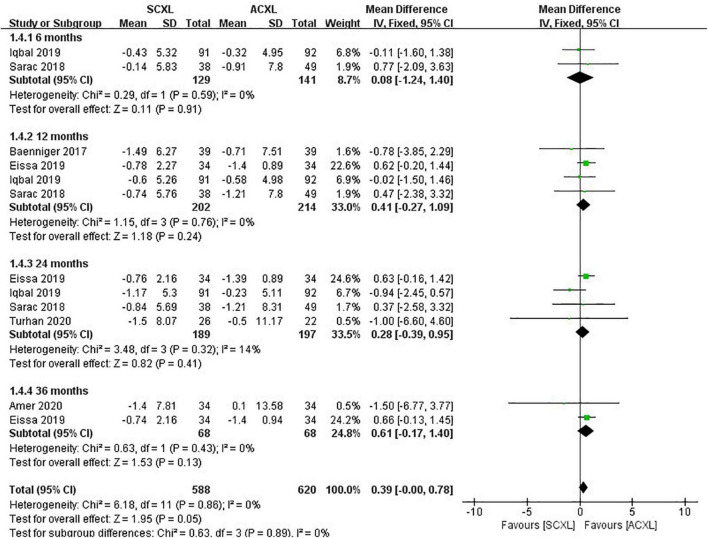
Forest plot of the change in Kmax (ΔKmax) of SCXL and ACXL.

Maximum keratometry (Kmax): The change in Kmax did not significantly differ between the two groups (WMD = 0.39, 95% CI: –0.00 to 0.78, *p* = 0.05, I^2^ = 0%), but it was likely that ACXL resulted in a greater decrease of Kmax than SCXL at 12-, 24-, and 36-month follow-up (*p* = 0.24, 0.41, and 0.13, respectively) (Figure 3).

#### Secondary Outcomes

**FIGURE 4:**
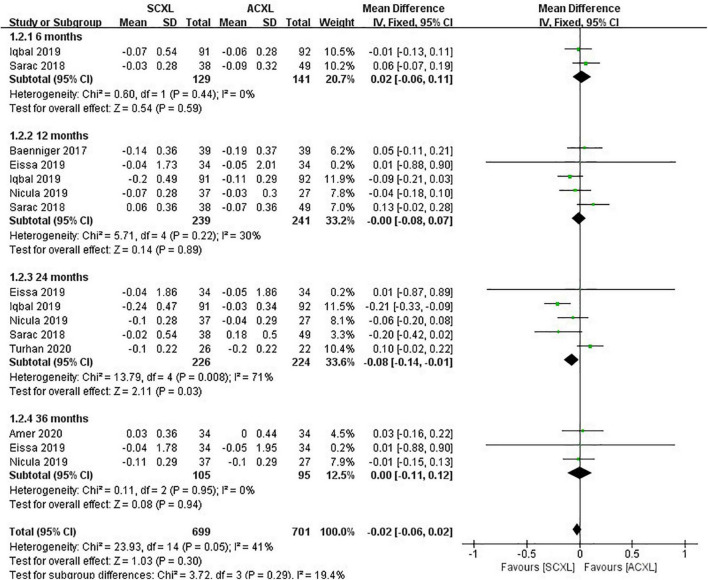
Forest plot of the change in BCVA (ΔBCVA) of SCXL and ACXL.

Best-corrected visual acuity. There were no significant differences for the change in BCVA (WMD = -0.02, 95% CI: –0.06 to 0.02, *p* = 0.30, I^2^ = 41%) between the two groups, but at the 24-month visit, the SCXL showed greater change in BCVA than ACXL (WMD = -0.08, 95% CI: –0.14 to –0.01, *p* = 0.03, I^2^ = 71%, Figure 4).

**FIGURE 5:**
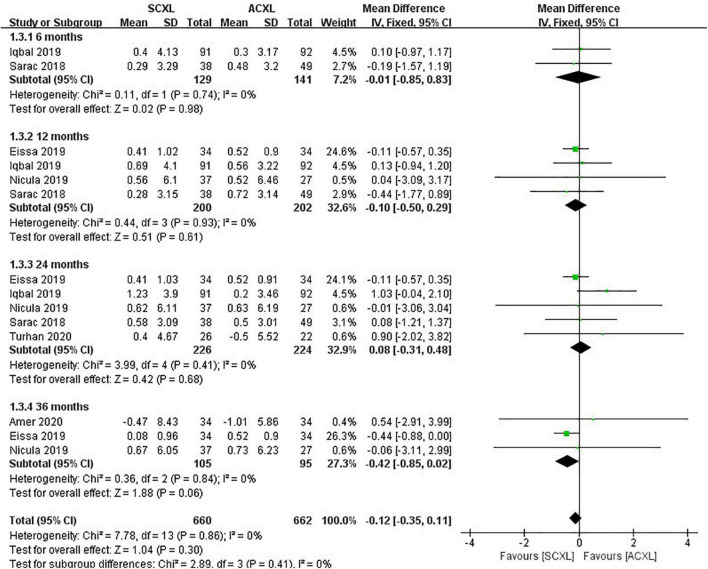
Forest plot of the change in MRSE (ΔMRSE) of SCXL and ACXL.

Manifest refraction: At the 6-, 12-, 36-, and 24-month visits, the changes in MRSE were comparable in both groups without significant difference (*p* = 0.98, 0.61, 0.06, and 0.68, respectively, Figure 5).

**FIGURE 6:**
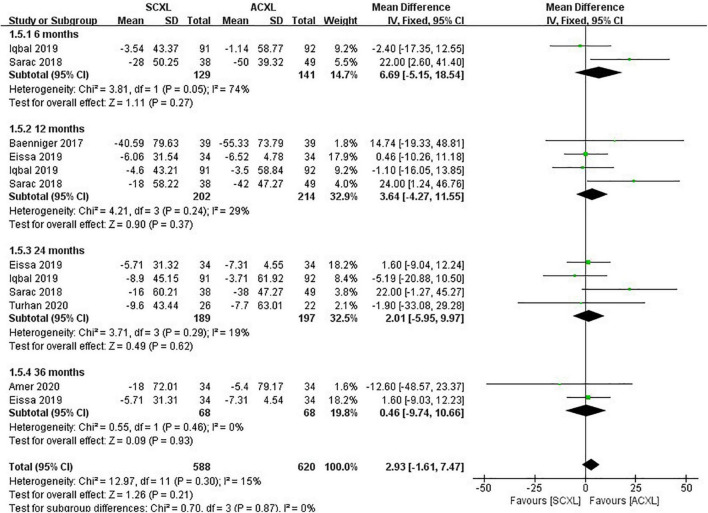
Forest plot of the change in CCT (ΔCCT) of SCXL and ACXL.

Central corneal thickness (CCT): At both short (6 and 12 months) and long-term (24 and 36 months) follow-ups, the reducing amounts of CCT in both groups were not significantly different (*p* = 0.27, 0.37, 0.62, and 0.93, respectively) (Figure 6).

#### Complications

The eye complications of CXL in both groups were analyzed. Common adverse effects include corneal haze, stromal infiltrates, opacity, persistent epithelial defect, delayed healing and pain, photophobia, and watery eyes. Haze formation is an important adverse event that occurs to threaten vision. The incidence of corneal haze was significantly increased in SCXL compared to ACXL [odds ratio (OR) 4.17; 95% CI 2.06–8.41, *p* < 0.0001] with high heterogeneity in the results (I^2^ = 73%, *p* = 0.03) (Supplementary Figure 2). When removing the source of heterogeneity (18), the two CXL procedures were comparable in terms of haze (OR 1.48, 95% CI 0.55–3.97, *p* = 0.43, I^2^ = 0%) (Supplementary Figure 3).

### Comparative Effectiveness of SCXL and Transepithelial CXL

#### Primary Outcomes

**FIGURE 7:**
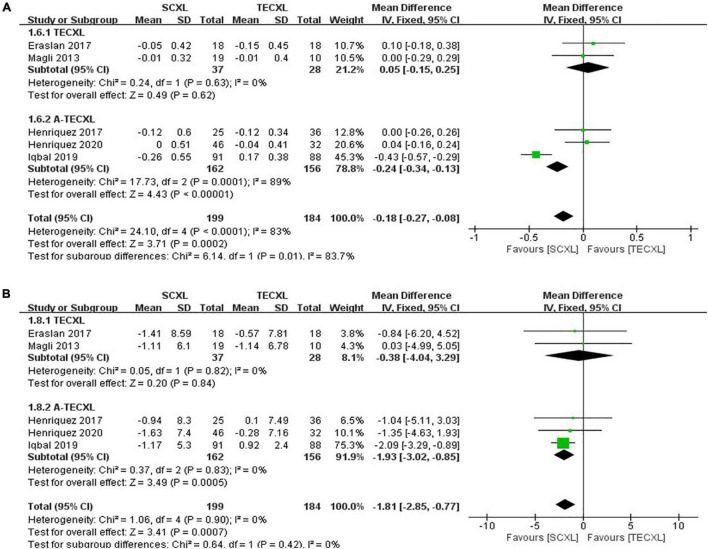
Forest plots of the changes in primary outcomes of SCXL and transepithelial CXL. **(A)** ΔUCVA, **(B)** ΔKmax.

Uncorrected visual acuity: There were totally five publications that involve the comparison between SCXL and transepithelial CXL (2 TECXL and 3 A-TECXL). Due to the small number of transepithelial CXL studies, we analyzed the TECXL and A-TECXL in subgroups and together. As shown in the Figure 7A, although in TECXL subgroup, the changes in UCVA after CXL were comparable between TECXL and SCXL (WMD = 0.05, 95%CI: –0.15 to 0.25, *p* = 0.62, I^2^ = 0%), the SCXL seemed to provide greater UCVA improvement than A-TECXL (WMD = -0.24, 95% CI: –0.34 to –0.13, *p* < 0.00001, I^2^ = 89%). However, the heterogeneities detected among studies were severe (*p* < 0.0001, I^2^ = 83%), the source of which was mostly from the A-TECXL subgroup.

Maximum keratometry: At final follow-up, SCXL may result in a greater change in Kmax after surgery than A-TECXL (WMD = -1.93, 95%CI: –3.02 to –0.85, *p* = 0.0005, I^2^ = 0%), whereas SCXL and TECXL were comparable in the change in Kmax post CXL (WMD = -0.38, 95% CI: –4.04 to 3.29, *p* = 0.84, I^2^ = 0%) (Figure 7B).

#### Secondary Outcomes

**FIGURE 8:**
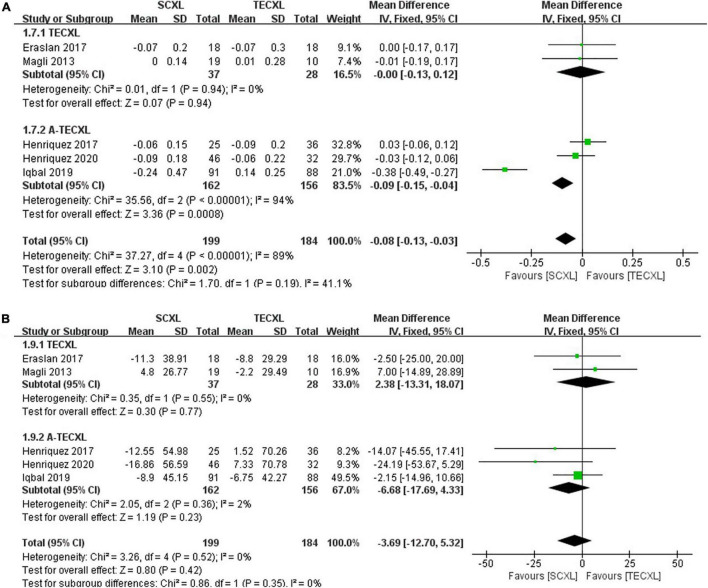
Forest plots of the changes in secondary outcomes of SCXL and transepithelial CXL. **(A)** ΔBCVA, **(B)** ΔCCT.

Best-corrected visual acuity: Subgroup results showed that SCXL was associated with better improvement in BCVA when compared to A-TECXL (WMD = -0.09, 95% CI: –0.15 to –0.04, *p* = 0.0008, I^2^ = 94%), whereas SCXL and TECXL were comparable in the change in BCVA after CXL (WMD = -0.00, 95% CI: –0.13 to 0.12, *p* = 0.94, I^2^ = 0%) (Figure 8A).

Central corneal thickness: Both pooling and subgroup results showed that changes in CCT before and after procedure in SCXL group and transepithelial CXL group at final follow-up were not statistically significant (pooling, WMD = -3.69, 95% CI: –12.7 to 5.32, *p* = 0.42, I^2^ = 0%, Figure 8B).

#### Complications

Among the five studies that compare SCXL and transepithelial CXL, all the reported corneal haze formation was observed in SCXL group. No haze was recorded in the TECXL or A-TECXL group (Supplementary Figure 4). Besides, eye pain and photophobia mostly occurred in the SCXL group.

#### Assessment of Study Quality and Publication Bias

The two RCTs were found to be of high quality. The Eissa et al. (19) study was found to have an unclear risk for the selection and detection bias, and the Iqbal et al. (18) study had an unclear risk of selection and performance bias (Supplementary Figure 1). According to NOS scores, the included non-RCT studies were of moderate-to-high quality and acceptable in the meta-analysis (Supplementary Table 2). The cohort studies performed less well mainly in their case definition of controls, method of ascertainment for cases and controls, and the non-response rate. Funnel plots for publication bias test for the outcomes in each pair of comparisons showed symmetric left-right distribution, which suggests no evidence of publication bias (Supplementary Figures 5, 6).

## Discussion

This meta-analysis evaluated and compared the efficacy of SCXL vs. ACXL and SCXL vs. TECXL for the treatment of keratoconus in pediatric patients. The results suggested that the changes in UCVA, BCVA, MRSE, Kmax, and CCT after surgery were not significantly different between SCXL and ACXL at 6-month, 1-, 2-, and 3-year follow-ups. One exception was that the change in BCVA after SCXL at 24-month visit was significantly greater than that after ACXL. The pooling results suggest that the standard epithelium-off and accelerated epithelium-off CXL protocols were comparable in the efficacy of pediatric keratoconus treatment. SCXL might result in greater improvement in UCVA, BCVA, and Kmax than A-TECXL, whereas in the comparison of SCXL and transepithelial CXL, no significant difference in the CCT reduction was found. Taken together, these indicate that in the treatment of pediatric keratoconus, SCXL might perform better in improving the visual outcomes than the transepithelial CXL, whereas SCXL and the accelerated CXL were similarly efficacious.

The previous researches mainly focused on the adult population, simply analyzed the postoperative outcomes in each CXL procedure (29), or only compared two or three CXL protocols (11, 13). It should be noted that Nath et al. (30) analyzed data of adult patients with mean age (SD) of 25.12 (8.83) years for transepithelial and 22.76 (9.06) years for epithelium-off, whereas our study only focused on the pediatric patients with keratoconus. Besides, Ng et al. (31) excluded studies that enrolled participants under the age of 14, and Ng et al. (32) also analyzed adult patients with mean age ranging from 23 to 30. Other differences between the studies and ours are the types of outcomes, the follow-up duration, and comparison pairs of CXL protocols. Indeed, the conclusions from our study show that for pediatric keratoconus, a significant greater increase in BCVA at 24-month follow-up in SCXL than ACXL was observed; SCXL seems to provide better visual and pachymetric outcomes than A-TECXL, with higher incidence of adverse events, agree with their results in some degrees. This suggests that for both adults and young patients, epithelium-off CXL could be considered as the standard treatment for progressive keratoconus, with superior efficacy to the safer transepithelial or perhaps accelerated CXLs.

Although recent prospective observational studies reported a comparable efficacy of conventional epithelium-off and accelerated CXL protocols in pediatric keratoconus management, there were also conflicting results from each study. Furthermore, small sample size, mild measured effects, and single-center design might undermine the conclusions. A meta-analysis pooling data from multiple studies may offer important insights into the comparison of different CXL techniques. Two meta-analyses analyzed children’s data and demonstrated the efficacy of SCXL, ACXL, and TECXL in preventing the progression of keratoconus (12, 14) and showed that all CXLs could attenuate the disease progression in the patients with pediatric keratoconus. These two studies were comprehensive, but not comparative for different CXL techniques. To our knowledge, the present report is the first to compare different CXL protocols in pediatric keratoconus by analyzing the changes in outcomes between pre- and postoperation.

A multicenter trial by Iqbal et al. showed that standard epithelium-off was more effective in pediatric keratoconus, attaining great stability as compared to either accelerated or transepithelial CXL (18). On the other hand, Eissa et al. reported that at 12-month follow-up, postoperative LogMAR UCVA, BCVA, and Kmax of accelerated CXL were statistically less than those of conventional CXL in pediatric keratoconus eyes (19). The conflicting results might result from varied follow-up duration and small sample sizes. Our meta-analysis could provide pooling data from multiple studies updated to 2020 and offer potential important insights into the CXL strategy prior to carrying out a large-scale clinical trial.

The included studies compared the efficacy and stability of either two or three of the standard epithelium-off, accelerated epithelium-off, transepithelial, and accelerated transepithelial CXL in pediatric keratoconus. An earlier meta-analysis by McAnena et al. has evaluated 13 papers from 2011 to 2014, which includes 490 eyes of 401 pediatric patients with keratoconus, which compared the pre- and postoperative CXL outcomes in standard epithelium-off and transepithelial protocol (22). However, they only analyzed the outcomes in either protocol but not between the two groups, likely due to the lack of enough published data at that time. The McAnena study found that standard protocol might be effective in halting progression of pediatric keratoconus, with significant improvement in UCVA and BCVA at 1 year and statistical reduction in Kmax at 2 years. In their results, no significant vision gain or change in Kmax was observed in the transepithelial group at 1-year visit. Besides, most of the included studies have multiple observing timepoints, which ranges from 12 to 48 months (Table 1), which allowed subgroup analysis according to the follow-ups. This meta-analysis compared postoperative outcomes between different CXL protocols in both short- and long terms.

Our results indicated that the long-term best spectacle-corrected visual outcomes (BCVA at 24 months) were in favor of SCXL as compared to ACXL (Figure 4). Furthermore, the changes in UCVA and BCVA in SCXL were significantly greater to those in A-TECXL group (Figures 7 and 8). These results are similar to those in Kobashi and Tsubota study, which focused on adult population and showed that ACXL has less effect on improving corrected visual acuity than SCXL after 1-year follow-up (33). The improvement in Kmax in SCXL was also significantly greater than those in A-TECXL group (Figure 7B). Soeters et al. reported that in adult population, transepithelial CXL might result in a continued keratoconus progression after 1 year (34). Ng et al. also showed that standard CXL resulted in a significantly greater reduction in Kmax and Kmean than its accelerated counterpart (35). Our results in pediatric patients are in accordance with these previous studies. No significant difference in UCVA, BCVA, and Kmax was found between the SCXL and TECXL group (Figures 7 and 8), but the small sample size suggests further clinical trials that compare that the two CXLs are required. The results indicated that in terms of halting progression and improving visual acuity, the standard epithelium-off CXL might be more efficacious than the others.

Corneal thickness was reported to increase after standard epithelium-off CXL possibly due to scattering formation, but stable CCT after CXL was also reported (36), possibly due to different measurement techniques such as the Orbscan II and Pentacam HR. Previously, it was shown that for adult patients with keratoconus, transepithelial CXL provided a more protective influence on corneal thickness than standard CXL (37). Early meta-analysis showed that accelerated CXL might lead to a less reduction in CCT than standard CXL (35), but recent studies suggest no difference between the ACXL and SCXL when comparing the CCT (16, 38). In pediatric population, our meta-analysis also observed no statistical difference in the change on postoperative CCT among different CXL protocols, regardless of different follow-up times (Figures 6 and 8).

Demarcation line (DL) is usually measured as a substitute indicator for the impact of CXL and treatment depth. The depths of DL typically range from 300 to 380 mm for SCXL and from 184 to 350 mm for ACXL (39), whereas for TECXL, the DLs are of approximately 90 to 110 mm in pediatric patients (37, 40). Although only Eraslan et al. reported the depths of DL among the included studies, their observation of shallower DLs after TECXL (137 mm) than those after SCXL (272 mm) was similar to the previous publications (22). A possible reason is the shorter soaking time with ACXL and the transepithelial process with TECXL as compared to SCXL. Our results of greater visual improvements SCXL than ACXL and A-TECXL suggest that SCXL might be more efficient than the other procedures, which is in accordance with the previous reported depths of DLs.

Endothelial cell density (ECD) was also an important factor that affects the recovery of pediatric patients. This meta-analysis did not enroll the ECD due to few records in the included studies. According to the previous reports, there was no statistically significant change in ECD between SCXL and ACXL (19) or between SCXL and TECXL in pediatrics (22, 23). Another trial also showed that counting of endothelial cell did not change significantly during follow-up in iontophoretic CXL (41). Besides, although being relatively rare, adverse events such as corneal edema, transient haze, permanent scar, sterile ulcer, and infectious keratitis should be recorded to provide more detailed observations of CXL complications in pediatrics. A recent study showed that 3 of 968 eyes developed infectious keratitis and seven sterile infiltrates after accelerated CXL over 4-year follow-up, but the studied population included both children and adults (42). Maharana et al. reported microbiological test results of the microbial keratitis after accelerated CXL, which shows mixed and simple infection in the cases (43). The mixed infections included coagulase-negative *Staphylococcus* (CoNS) with *Aspergillus fumigatus*, *Staphylococcus aureus* with *Mucor* spp., *S. aureus* with *Acanthamoeba*, and simple infection included *S. aureus*, CoNS, and *Alternaria* spp. (43).

There are some limitations in this meta-analysis. First, the surgical procedures of CXL in each protocol were not exactly the same. In the epithelium-off groups, the process of epithelium removal was different in concentration of ethanol and soaking time, and the riboflavin instilment interval during UVA exposure was not uniform. In the transepithelial groups, the concentrations of riboflavin in soaking, the UVA power, and the process of UVA exposure were varied. Second, Kmax is often measured with different measurements, such as Pentacam, Precisio, and Sirius. Different measurement system was confounding factor that might introduce inconsistency in the value of Kmax. In this meta-analysis, however, we compared the difference between pre- and postoperative parameters (the “Δ,” post–pre), not the absolute value. To some degrees, this would reduce the confounding brought by different measurements. Third, due to the scarcely reported RCTs, there were only 2 RCTs included whereas the others were of retrospective or prospective comparative design, which would increase the risk of potential selection and publication bias. Fourth, some outcomes had high heterogeneity, such as the Kmax in subgroup analysis between SCXL and ACXL, and the UCVA and BCVA between SCXL and A-TECXL, which were possibly caused by different baseline features, surgical techniques, the inclusion of both RCTs and CNSs, and also the diversity in ethnicity. The evaluated studies were carried out in Europe, North Africa, Western Asia, and South America, and thus, effects in other regions such as East Asian remain unclear. We encourage researchers in different countries or races to conduct more RCTs to provide specific data and results in details.

## Conclusion

In summary, for pediatric keratoconus, except for a significant greater increase in BCVA at 24-month follow-up in SCXL than ACXL, both SCXL and ACXL are comparable on visual effects and keratometric outcomes; the incidence of postoperative corneal haze may be higher in the SCXL than ACXL, but the conclusion should be appreciated with caution due to severe heterogeneity. SCXL seems to provide better visual and pachymetric outcomes than A-TECXL, with higher incidence of adverse events. Larger RCTs in longer follow-up terms with complete panels of parameters including ECD are necessary to evaluate the efficacy and safety of CXL procedures for pediatric patients.

## Data Availability Statement

The original contributions presented in the study are included in the article/Supplementary Material, further inquiries can be directed to the corresponding author.

## Author Contributions

YLi, XW, and DW: research design. YLi, KD, YY, and TH: data acquisition, research execution, and data analysis. YLu, YF, AX, QF, and DW: manuscript preparation. All authors contributed to the article and approved the submitted version.

## Conflict of Interest

The authors declare that the research was conducted in the absence of any commercial or financial relationships that could be construed as a potential conflict of interest.

## Publisher’s Note

All claims expressed in this article are solely those of the authors and do not necessarily represent those of their affiliated organizations, or those of the publisher, the editors and the reviewers. Any product that may be evaluated in this article, or claim that may be made by its manufacturer, is not guaranteed or endorsed by the publisher.
