# Supplementary material for: Comparison of Efficacy and Safety Between Standard, Accelerated Epithelium-Off and Transepithelial Corneal Collagen Crosslinking in Pediatric Keratoconus: A Meta-Analysis
Source: Front Med (Lausanne). 2022 Mar 17;9:787167. doi: 10.3389/fmed.2022.787167 (PMC8968048; doi:10.3389/fmed.2022.787167)
Supplement: Supplementary file 1 [file Data_Sheet_1.docx]

**Supplementary Material**

**Comparison of efficacy and safety between standard, accelerated epithelium-off and transepithelial corneal collagen cross-linking in pediatric keratoconus: a meta-analysis**

***Yuanjun Li^1^, Ying Lu^1^, Kaixuan Du^1^, Yewei Yin^1^, Tu Hu^1^, Yanyan Fu^1^, Aiqun Xiang^1^, Qiuman Fu^1^, Xiaoying Wu^1^, Dan Wen^1*^***

1 Eye Center of Xiangya Hospital, Hunan Key Laboratory of Ophthalmology, National Clinical Research Center for Geriatric Disorders, Xiangya Hospital, Central South University, Changsha 410008, Hunan Province, China.

*** Correspondence:**

Dan Wen

wendan@csu.edu.cn

- ***Supplementary Online Content List***

**Supplementary Figure 1.** Quality assessment of RCTs included in the meta-analysis According to the Cochrane Collaboration Tool.

**Supplementary Figure 2.** Forest plots of the incidence of corneal haze in SCXL compared to ACXL.

**Supplementary Figure 3.** Forest plots of the incidence of corneal haze in SCXL compared to ACXL removing the source of heterogeneity.

**Supplementary Figure 4.** Forest plots of the incidence of corneal haze in SCXL compared to transepithelial CXL.

**Supplementary Figure 5.** Funnel plots for publication bias for different outcomes in the comparison between SCXL vs ACXL.

**Supplementary Figure 6.** Funnel plots for publication bias for different outcomes in the comparison between SCXL vs TECXL.

**Supplementary Table 1.** Searching strategy.

**Supplementary Table 2.** Quality assessment of non-RCTs included in the meta-analysis using Newcastle-Ottawa Scale.

**Supplementary Table 3.** Surgical procedures of the included studies in the meta-analysis.


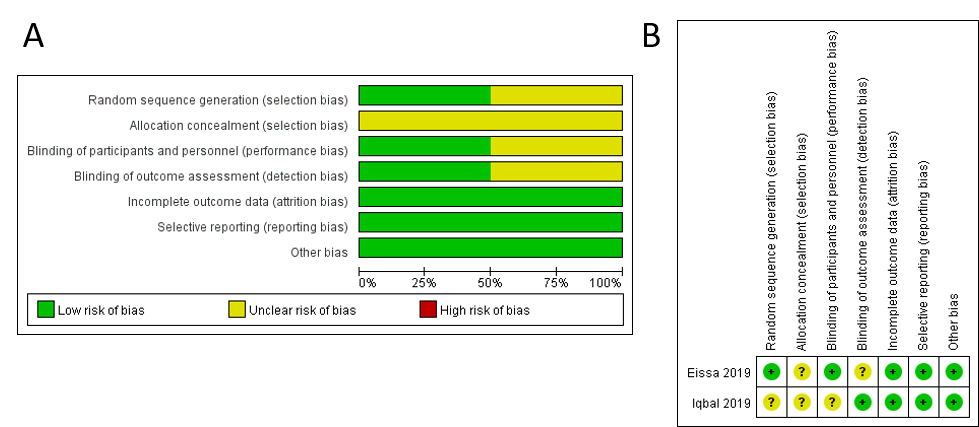


**Supplementary Figure 1. Quality assessment of RCTs included in the meta-analysis According to the Cochrane Collaboration Tool.** A. Risk of bias in individual trials. risk of bias item presented as percentages across included studies. B. Risk of bias across included trials weighted by sample sizes.

**
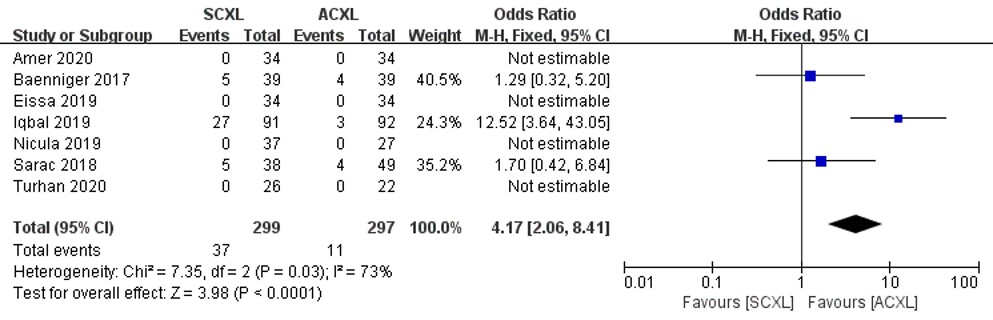
**

**Supplementary Figure 2. Forest plots of the incidence of corneal haze in SCXL compared to ACXL.**

**
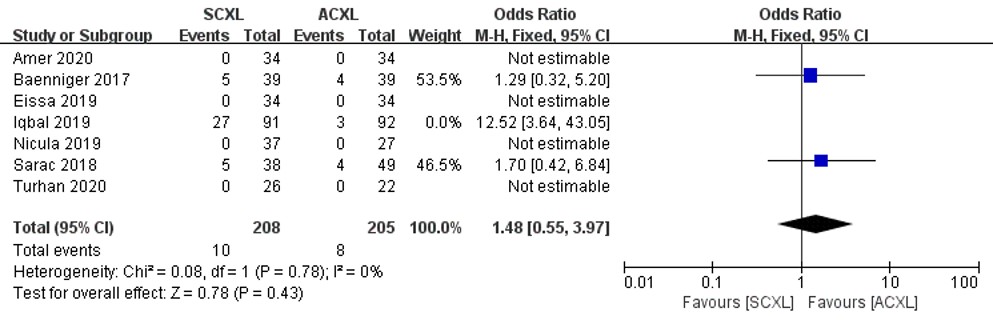
**

**Supplementary Figure 3. Forest plots of the incidence of corneal haze in SCXL compared to ACXL removing the source of heterogeneity.**

**
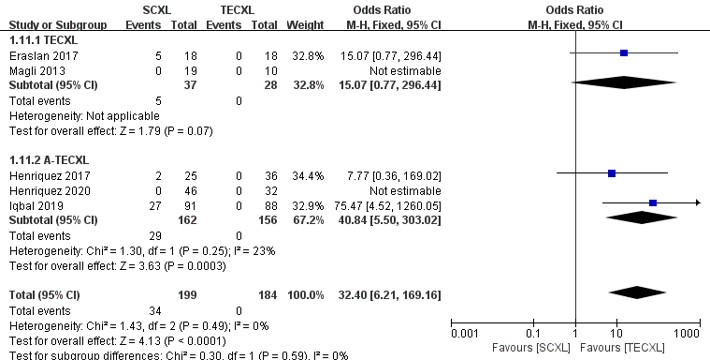
**

**Supplementary Figure 4. Forest plots of the incidence of corneal haze in SCXL compared to transepithelial CXL.**


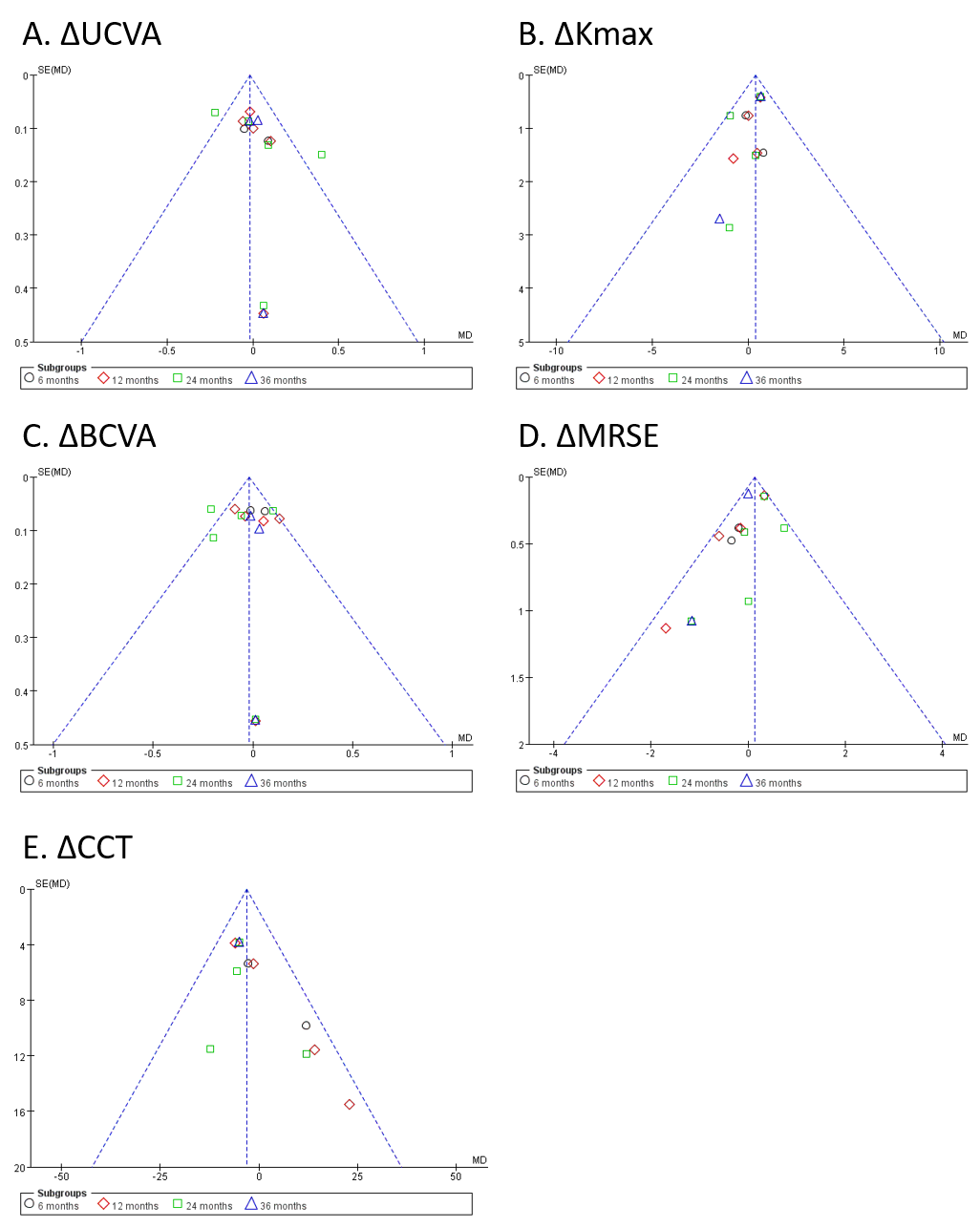


**Supplementary Figure 5. Funnel plots for publication bias for different outcomes in the comparison between SCXL vs ACXL.** A. ΔUCVA, B. ΔKmax, C. ΔBCVA, D. ΔMRSE, E. ΔCCT.


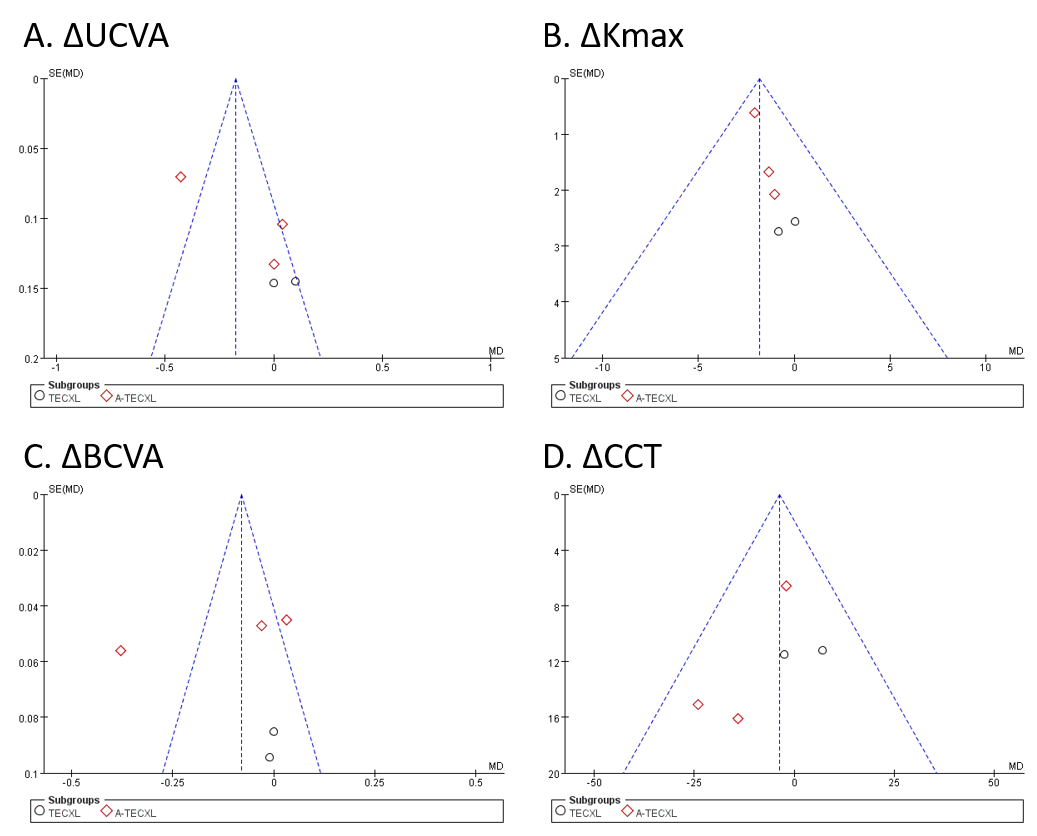


**Supplementary Figure 6. Funnel plots for publication bias for different outcomes in the comparison between SCXL vs TECXL.** A. ΔUCVA, B. ΔKmax, C. ΔBCVA, D. ΔCCT.

**Supplementary Table 1. Searching strategy**

**Number of citations by database searched**

| **Databases** | **Citations** |
| --- | --- |
| PubMed | 79 |
| EMBASE | 94 |
| Cochrane Library | 6 |
| ClinicalTrials.gov | 84 |
| Total (with duplicates) | 263 |

| Electronic databases searched | PubMed (http://www.ncbi.nlm.nih.gov/PubMed/),  EMBASE (http://www.embase.com),  Cochrane Library (http://www.thecochranelibrary.com/), ClinicalTrials.gov (https://clinicaltrials.gov/) |
| --- | --- |
| Search terms for PubMed was conducted to identify qualified literature from inception to March, 2021(Similar search strategies were modified according to the requirements of EMBASE and Cochrane Library databases). | 1. “Keratoconus” [MeSH Terms] 2. “Corneal” [MeSH Terms] 3. “Collagen” [MeSH Terms] 4. “Cross-linking reagents” [MeSH Terms] 5. “Epithelium, corneal” [MeSH Terms] 6. “Debridement” [MeSH Terms] 7. “Adolescent” [MeSH Terms] 8. “Child” [MeSH Terms] 9. “Young adult” [MeSH Terms] 10. Corneal collagen cross linking [Title/Abstract] 11. CXL [Title/Abstract] 12. Pediatric [Title/Abstract] 13. Epithelium off [Title/Abstract] 14. Epithelium on [Title/Abstract] 15. Standard [Title/Abstract] 16. Mechanical [Title/Abstract] 17. Accelerated [Title/Abstract] 18. Transepithelial [Title/Abstract] 19. 1 OR 2 OR 3 OR 4 OR 5 OR 6 OR 7 OR 8 OR 9 OR 10 OR 11 OR 12 OR 13 OR 14 OR 15 OR 16 OR 17 OR 18 20. “Follow-up studies” [MeSH Terms] 21. “Retrospective studies” [MeSH Terms] 22. “Prospective studies” [MeSH Terms] 23. “Treatment outcome” [MeSH Terms] 24. Effect [Title/Abstract] 25. Efficacy [Title/Abstract] 26. 20 OR 21 OR 22 OR 23 OR 24 OR 25 27. 19 AND 26 |
| Search terms for ClinicalTrials.gov | Condition or disease:  Keratoconus AND cross linking  (With or without) Other terms:  Pediatric OR young AND Epithelium off OR Epithelium on OR Standard OR Mechanical OR Accelerated OR Transepithelial  (With or without) Applied filters:  Child (birth-17)*  *Any relevant trials of patients at the age of 18 or younger were also reviewed and identified. |
| Limitation | Articles written in English were considered eligible. |
| Other sources | The references of included articles were searched and reviewed to recognize any relevant literatures. |

**Supplementary Table 2. Quality assessment of non-RCTs included in the meta-analysis using Newcastle-Ottawa Scale**

| Methodological  Item For Nonrandomized  Studies (No. 1-8) | 1. Is the Case Definition Adequate? | 2. Representativeness of the Cases | 3. Selection of Controls | 4. Definition of Controls | 5. Comparability of Cases and Controls on the Basis of the Design or Analysis | 6. Ascertainment of Exposure | 7. Same method of ascertainment for cases and controls | 8. Non-Response Rate | Total NOS Score |
| --- | --- | --- | --- | --- | --- | --- | --- | --- | --- |
| Baenninger 2017 | 1 | 1 | 1 | 1 | 2 | 1 | 0 | 0 | 7 |
| Nicula 2019 | 1 | 1 | 1 | 1 | 1 | 1 | 1 | 0 | 7 |
| Sarac 2018 | 1 | 1 | 1 | 0 | 1 | 1 | 1 | 0 | 6 |
| Turhan 2020 | 1 | 1 | 1 | 1 | 2 | 1 | 0 | 0 | 7 |
| Eraslan 2017 | 1 | 1 | 1 | 1 | 2 | 1 | 0 | 0 | 7 |
| Henriquez 2017 | 1 | 1 | 1 | 1 | 2 | 1 | 0 | 0 | 7 |
| Henriquez 2020 | 1 | 1 | 1 | 1 | 2 | 1 | 0 | 0 | 7 |
| Amer 2020 | 1 | 1 | 1 | 0 | 1 | 1 | 1 | 0 | 6 |
| Magli 2013 | 1 | 1 | 1 | 1 | 2 | 1 | 0 | 0 | 7 |

The selection area included No. 1–4, which was up to one score in one question; The comparability area included No. 5, which was up to 2 scores in the question; The exposure area included No. 6–8, which was up to one score in one question. The total score was 9.

**Supplementary Table 3. Surgical procedures of the included studies in the meta-analysis.**

|  | **Reference** | **Epithelium removal** | **Riboflavin soaking** | | **UVA exposure** | | |
| --- | --- | --- | --- | --- | --- | --- | --- |
|  |  |  | **Riboflavin conc.** | **Duration/ Ribo. instilment frequency (min/min)** | **Power (mW/cm^2^)** | **Duration/Ribo. instilment frequency (min/min)** | **Total Energy (J/cm^2^)** |
| (a) SCXL | Baenninger 2017 | 20% ethanol 10 s | 0.1% | 30/3 | 3.0 | 30/3 | NG |
|  | Eissa 2019 | 50% alcohol 20 s | 0.1% | 30/NG | 3.0 | 30/5 | NG |
|  | Eraslan 2017 | Mechanical | 0.1% | 30/NG | 3.0 | 30/2 | NG |
|  | Henriquez 2017 | Mechanical | 0.1% | 30/NG | 3.0 | 30/5 | NG |
|  | Iqbal 2019 | Mechanical | 0.1% | 30/3 | 3.0 | 30/2 | 5.4 |
|  | Magli 2013 | Mechanical | 0.1% | 30/NG | 3.0 | 30/2 | NG |
|  | Nicula 2019 | NG | 0.1% | 30/3 | 3.0 | 30/3 | NG |
|  | Sarac 2018 | Mechanical + 20% alcohol 20 s | 0.1% | 30/3 | 3.0 | 30/NG | 5.4 |
|  | Henriquez 2020 | Mechanical | 0.1% | 30/5 | 3.0 | 30/5 | NG |
|  | Amer 2020 | Mechanical | 0.1% | 30/3 | 3.0 | 30/NG | 5.4 |
|  | Turhan 2020 | Mechanical | 0.1% | 30/2 | 3.0 | 30/NG | 5.4 |
| (b) ACXL | Baenninger 2017 | 20% alcohol 10 s | 0.1% | 30/NG | 9.0 | 10/NG | NG |
|  | Eissa 2019 | 50% alcohol 20 s | 0.1% | 30/NG | 18.0 | 5/NG | 5.4 |
|  | Iqbal 2019 | Mechanical | 0.1% | 10/2 | 30.0 | 8 min of pulsed  mode* | 7.2 |
|  | Nicula 2019 | NG | 0.1% | 20/2 | 9.0 | 10/NG | NG |
|  | Turhan 2020 | Mechanical | 0.1% | 20/2 | 9.0 | 10/NG | 5.4 |
|  | Amer 2020 | Mechanical | 0.1% | 30/3 | 9.0 | 10/NG | 5.4 |
|  | Sarac 2018 | Mechanical | 0.1% | 30/3 | 9.0 | 10/NG | 5.4 |
| (c) TECXL | Eraslan 2017 | - | 0.25% | 30/2 | 3.0 | 30/2 | NG |
|  | Magli 2013 | - | 0.1% | 30/5 | 3.0 | 30/NG | NG |
| (d) A-TECXL | Henriquez 2017 | Mechanical | 0.25% | 30/5 | 18.0 | 5/NG | NG |
|  | Henriquez 2020 | Mechanical | 0.25% | 30/5 | 18.0 | 5/NG | NG |
|  | Iqbal 2019 | - | 0.22% | 6/1.5 | 45.0 | 40 min of pulsed mode | 7.2 |

**Reference**

1. Krachmer JH, Feder RS, Belin MW. Keratoconus and related noninflammatory corneal thinning disorders. Surv Ophthalmol. 1984;28(4):293-322.

2. Mukhtar S, Ambati BK. Pediatric keratoconus: a review of the literature. Int Ophthalmol. 2018;38(5):2257-66.

3. Olivo-Payne A, Abdala-Figuerola A, Hernandez-Bogantes E, Pedro-Aguilar L, Chan E, Godefrooij D. Optimal management of pediatric keratoconus: challenges and solutions. Clin Ophthalmol. 2019;13:1183-91.

4. Tuft SJ, Moodaley LC, Gregory WM, Davison CR, Buckley RJ. Prognostic factors for the progression of keratoconus. Ophthalmology. 1994;101(3):439-47.

5. Leoni-Mesplie S, Mortemousque B, Touboul D, Malet F, Praud D, Mesplie N, et al. Scalability and severity of keratoconus in children. Am J Ophthalmol. 2012;154(1):56-62 e1.

6. El Rami H, Chelala E, Dirani A, Fadlallah A, Fakhoury H, Cherfan C, et al. An Update on the Safety and Efficacy of Corneal Collagen Cross-Linking in Pediatric Keratoconus. Biomed Res Int. 2015;2015:257927.

7. Sinha Roy A, Shetty R, Kummelil MK. Keratoconus: a biomechanical perspective on loss of corneal stiffness. Indian J Ophthalmol. 2013;61(8):392-3.

8. Sharif R, Sejersen H, Frank G, Hjortdal J, Karamichos D. Effects of collagen cross-linking on the keratoconus metabolic network. Eye (Lond). 2018;32(7):1271-81.

9. Brooks NO, Greenstein S, Fry K, Hersh PS. Patient subjective visual function after corneal collagen crosslinking for keratoconus and corneal ectasia. J Cataract Refract Surg. 2012;38(4):615-9.

10. Wollensak G, Spoerl E, Seiler T. Stress-strain measurements of human and porcine corneas after riboflavin-ultraviolet-A-induced cross-linking. J Cataract Refract Surg. 2003;29(9):1780-5.

11. Fard AM, Reynolds AL, Lillvis JH, Nader ND. Corneal collagen cross-linking in pediatric keratoconus with three protocols: a systematic review and meta-analysis. J AAPOS. 2020;24(6):331-6.

12. McAnena L, O'Keefe M. Corneal collagen crosslinking in children with keratoconus. J AAPOS. 2015;19(3):228-32.

13. Wen D, Song B, Li Q, Tu R, Huang Y, Wang Q, et al. Comparison of Epithelium-Off Versus Transepithelial Corneal Collagen Cross-Linking for Keratoconus: A Systematic Review and Meta-Analysis. Cornea. 2018;37(8):1018-24.

14. Kobashi H, Hieda O, Itoi M, Kamiya K, Kato N, Shimazaki J, et al. Corneal Cross-Linking for Paediatric Keratoconus: A Systematic Review and Meta-Analysis. J Clin Med. 2021;10(12).

15. Higgins JP, Altman DG, Gotzsche PC, Juni P, Moher D, Oxman AD, et al. The Cochrane Collaboration's tool for assessing risk of bias in randomised trials. BMJ. 2011;343:d5928.

16. Wen D, Li Q, Song B, Tu R, Wang Q, O'Brart DPS, et al. Comparison of Standard Versus Accelerated Corneal Collagen Cross-Linking for Keratoconus: A Meta-Analysis. Invest Ophthalmol Vis Sci. 2018;59(10):3920-31.

17. Stang A. Critical evaluation of the Newcastle-Ottawa scale for the assessment of the quality of nonrandomized studies in meta-analyses. Eur J Epidemiol. 2010;25(9):603-5.

18. Iqbal M, Elmassry A, Saad H, Am Gad A, Ibrahim O, Hamed N, et al. Standard cross-linking protocol versus accelerated and transepithelial cross-linking protocols for treatment of paediatric keratoconus: a 2-year comparative study. Acta Ophthalmol. 2020;98(3):e352-e62.

19. Eissa SA, Yassin A. Prospective, randomized contralateral eye study of accelerated and conventional corneal cross-linking in pediatric keratoconus. Int Ophthalmol. 2019;39(5):971-9.

20. Nicula CA, Rednik AM, Bulboaca AE, Nicula D. Comparative Results Between "Epi-Off" Conventional and Accelerated Corneal Collagen Crosslinking for Progressive Keratoconus in Pediatric Patients. Ther Clin Risk Manag. 2019;15:1483-90.

21. Baenninger PB, Bachmann LM, Wienecke L, Thiel MA, Kaufmann C. Pediatric Corneal Cross-linking: Comparison of Visual and Topographic Outcomes Between Conventional and Accelerated Treatment. Am J Ophthalmol. 2017;183:11-6.

22. Eraslan M, Toker E, Cerman E, Ozarslan D. Efficacy of Epithelium-Off and Epithelium-On Corneal Collagen Cross-Linking in Pediatric Keratoconus. Eye Contact Lens. 2017;43(3):155-61.

23. Magli A, Forte R, Tortori A, Capasso L, Marsico G, Piozzi E. Epithelium-off corneal collagen cross-linking versus transepithelial cross-linking for pediatric keratoconus. Cornea. 2013;32(5):597-601.

24. Henriquez MA, Rodriguez AM, Izquierdo L, Jr. Accelerated Epi-On Versus Standard Epi-Off Corneal Collagen Cross-Linking for Progressive Keratoconus in Pediatric Patients. Cornea. 2017;36(12):1503-8.

25. Henriquez MA, Hernandez-Sahagun G, Camargo J, Izquierdo L, Jr. Accelerated Epi-On Versus Standard Epi-Off Corneal Collagen Cross-Linking for Progressive Keratoconus in Pediatric Patients: Five Years of Follow-Up. Cornea. 2020;39(12):1493-8.

26. Sarac O, Caglayan M, Uysal BS, Uzel AGT, Tanriverdi B, Cagil N. Accelerated versus standard corneal collagen cross-linking in pediatric keratoconus patients: 24 months follow-up results. Cont Lens Anterior Eye. 2018;41(5):442-7.

27. Turhan SA, Yargi B, Toker E. Efficacy of Conventional Versus Accelerated Corneal Cross-linking in Pediatric Keratoconus: Two-Year Outcomes. J Refract Surg. 2020;36(4):265-9.

28. Amer I, Elaskary A, Mostafa A, Hazem HA, Omar A, Abdou A. Long-Term Visual, Refractive and Topographic Outcomes of "Epi-off" Corneal Collagen Cross-Linking in Pediatric Keratoconus: Standard versus Accelerated Protocol. Clin Ophthalmol. 2020;14:3747-54.

29. Tian M, Jian W, Sun L, Shen Y, Zhang X, Zhou X. One-year follow-up of accelerated transepithelial corneal collagen cross-linking for progressive pediatric keratoconus. BMC Ophthalmol. 2018;18(1):75.

30. Nath S, Shen C, Koziarz A, Banfield L, Nowrouzi-Kia B, Fava MA, et al. Transepithelial versus Epithelium-off Corneal Collagen Cross-linking for Corneal Ectasia: A Systematic Review and Meta-analysis. Ophthalmology. 2021;128(8):1150-60.

31. Ng SM, Hawkins BS, Kuo IC. Transepithelial Versus Epithelium-Off Corneal Crosslinking for Progressive Keratoconus: Findings From a Cochrane Systematic Review. Am J Ophthalmol. 2021;229:274-87.

32. Ng SM, Ren M, Lindsley KB, Hawkins BS, Kuo IC. Transepithelial versus epithelium-off corneal crosslinking for progressive keratoconus. Cochrane Database Syst Rev. 2021;3:CD013512.

33. Kobashi H, Tsubota K. Accelerated Versus Standard Corneal Cross-Linking for Progressive Keratoconus: A Meta-Analysis of Randomized Controlled Trials. Cornea. 2020;39(2):172-80.

34. Soeters N, Wisse RP, Godefrooij DA, Imhof SM, Tahzib NG. Transepithelial versus epithelium-off corneal cross-linking for the treatment of progressive keratoconus: a randomized controlled trial. Am J Ophthalmol. 2015;159(5):821-8 e3.

35. Ng AL, Chan TC, Cheng AC. Conventional versus accelerated corneal collagen cross-linking in the treatment of keratoconus. Clin Exp Ophthalmol. 2016;44(1):8-14.

36. Kaya F. Epithelium-off corneal cross-linking in progressive keratoconus: 6- year outcomes. J Fr Ophtalmol. 2019;42(4):375-80.

37. Filippello M, Stagni E, O'Brart D. Transepithelial corneal collagen crosslinking: bilateral study. J Cataract Refract Surg. 2012;38(2):283-91.

38. Shajari M, Kolb CM, Agha B, Steinwender G, Muller M, Herrmann E, et al. Comparison of standard and accelerated corneal cross-linking for the treatment of keratoconus: a meta-analysis. Acta Ophthalmol. 2019;97(1):e22-e35.

39. Bouheraoua N, Jouve L, El Sanharawi M, Sandali O, Temstet C, Loriaut P, et al. Optical coherence tomography and confocal microscopy following three different protocols of corneal collagen-crosslinking in keratoconus. Invest Ophthalmol Vis Sci. 2014;55(11):7601-9.

40. Buzzonetti L, Petrocelli G. Transepithelial corneal cross-linking in pediatric patients: early results. J Refract Surg. 2012;28(11):763-7.

41. Buzzonetti L, Petrocelli G, Valente P, Iarossi G, Ardia R, Petroni S. Iontophoretic transepithelial corneal cross-linking to halt keratoconus in pediatric cases: 15-month follow-up. Cornea. 2015;34(5):512-5.

42. Kodavoor SK, Tiwari NN, Ramamurthy D. Profile of infectious and sterile keratitis after accelerated corneal collagen cross-linking for keratoconus. Oman J Ophthalmol. 2020;13(1):18-23.

43. Maharana PK, Sahay P, Sujeeth M, Singhal D, Rathi A, Titiyal JS, et al. Microbial Keratitis After Accelerated Corneal Collagen Cross-Linking in Keratoconus. Cornea. 2018;37(2):162-7.

44. Li YJ, Yin YW, Hu T, Du KX, Lu Y, Fu YY, et al. Comparison of Standard Versus Accelerated Epithelium-Off and Transepithelial Corneal Cross-Linking in Pediatric Keratoconus: An Up-to-date Meta-Analysis. Research Square [Preprint] 2020. Available at: https://doi.org/10.21203/rs.3.rs-29160/v1 (Accessed May 29, 2020).
